# β1-blocker in sepsis

**DOI:** 10.1186/s40560-021-00552-w

**Published:** 2021-05-08

**Authors:** Daisuke Hasegawa, Ryota Sato, Osamu Nishida

**Affiliations:** 1 Department of Anesthesiology and Critical Care Medicine, Fujita Health University School of Medicine, 1-98 Dengakugakubo, Kutsukake-cho, Toyoake, Aichi 470-1192 Japan; 2 Department of Critical Care Medicine, Respiratory Institute, Cleveland Clinic, Cleveland, OH USA

**Keywords:** Sepsis, Ultrashort-acting β1-blockers, Esmolol, Landiolol, Non-compensatory tachycardia, Persistent tachycardia

## Abstract

**Background:**

The use of ultrashort-acting β1-blockers recently has attracted attention in septic patients with non-compensatory tachycardia. We summarized the metabolic and hemodynamic effects and the clinical evidence of ultrashort-acting β1-blockers.

**Main body:**

A recent meta-analysis showed that ultrashort-acting β1-blockers reduced the mortality in septic patients with persistent tachycardia. However, its mechanism to improve mortality is not fully understood yet. We often use lactate as a marker of oxygen delivery, but an impaired oxygen use rather than reduced oxygen delivery has been recently proposed as a more reasonable explanation of hyperlactatemia in patients with sepsis, leading to a question of whether β1-blockers affect metabolic systems. While the stimulation of the β2-receptor accelerates glycolysis and lactate production, the role of β1-blocker in lactate production remains unclear and studies investigating the role of β1-blockers in lactate kinetics are warranted. A meta-analysis also reported that ultrashort-acting β1-blockers increased stroke volume index, while it reduced heart rate, resulting in unchanged cardiac index, mean arterial pressure, and norepinephrine requirement at 24 h, leading to an improvement of cardiovascular efficiency. On the other hand, a recent study reported that heart rate reduction using fast esmolol titration in the very early phase of septic shock caused hemodynamic instability, suggesting that ultrashort-acting β1-blockers should be started only after completing initial resuscitation. While many clinicians still do not feel comfortable controlling sinus tachycardia, one randomized controlled trial in which the majority had sinus tachycardia suggested the mortality benefit of ultrashort-acting β1-blockers. Therefore, it still deems to be reasonable to control sinus tachycardia with ultrashort-acting β1-blockers after completing initial resuscitation.

**Conclusion:**

Accumulating evidence is supporting the use of ultrashort-acting β1-blockers while larger randomized controlled trials to clarify the effect of ultrashort-acting β1-blockers are still warranted.

## Background

In patients with sepsis, the adrenergic system serves as an initial adaptive response to maintain homeostasis. However, excessive catecholamine surge can cause adverse effects such as persistent tachycardia, which worsens the prognosis in patients with sepsis [1]. Therefore, the use of β-blockers in sepsis recently has attracted attention especially in patients with tachycardia.

Tachycardia seen in sepsis is usually secondary to hypovolemia, fever, or pain. However, even without these conditions, we often encounter persistent tachycardia, which is likely to be non-compensatory tachycardia due to sympathetic overstimulation [2].

While our recent meta-analysis suggested that the use of ultrashort-acting β1-blockers improved mortality in septic patients with non-compensatory tachycardia [3], many clinicians still believe that sinus tachycardia should not be controlled with medications. This conflict between evidence and experience raises the question of whether β1-blockers should be used in septic patients with non-compensatory sinus tachycardia. In addition to heart rate (HR) reduction, ultrashort-acting β1-blockers were also associated with significantly reduced white blood cell counts in this meta-analysis [3]. Although various effects including coagulation and immunological modifications were reported more than a decade ago [4], clinical evidence supporting these effects in actual clinical settings is still very limited. Of these various effects, the oxygen utilization has been focused on given that β-stimulants such as epinephrine are well-known to increase lactate level [5], implying that β1-blockers may, in contrast, improve the oxygen utilization in patients with sepsis.

Hereby, we summarized the metabolic and hemodynamic effects and the clinical evidence of β1-blockers in patients with sepsis.

## Metabolic effects

We often use lactate as a marker of oxygen delivery. However, Morelli et al. reported that esmolol was associated with a significant reduction in lactate in patients with sepsis though it also reduced oxygen delivery [6]. Further, another study has shown that hyperlactatemia in sepsis was mainly caused by impaired tissue oxygen use rather than by reduced oxygen delivery [7]. Therefore, an impaired oxygen use has been proposed as a more reasonable explanation of hyperlactatemia in sepsis. It is well-known that the stimulation of the β2-receptor increases the production of cyclic adenosine monophosphate, accelerating glycolysis [5]. Therefore, the β2-blockade is likely to result in a reduction of lactate production. On the other hand, the patients’ ability to accelerate glycolysis and lactate production in response to epinephrine administration is reported to be associated with a better prognosis [8]. This suggests that an accelerated glycolysis and lactate production may be an adaptive response. Further, the role of β1-blocker in lactate production, unlike β2-blockade, remains unclear. Therefore, suppression of lactate production does not fully explain the mortality-benefit of β1-blocker in patients with sepsis.

Besides lactate level itself, lactate clearance is known to be a strong predictor of mortality in patients with sepsis [9]. Although a recent meta-analysis described that ultrashort-acting β1-blockers improved lactate clearance at 72 h [3], lactate clearance is a dynamic and complicated process since it involves both lactate production and metabolization. Therefore, studies investigating the role of β1-blocker in lactate kinetics are still warranted.

## Hemodynamic effects

Our recent meta-analysis also reported that the use of ultrashort-acting β1-blockers increased stroke volume index (SVI), while it reduced HR, resulting in unchanged cardiac index (CI), mean arterial pressure (MAP), and norepinephrine requirement at 24 h [3]. Especially, HR was successfully reduced with ultrashort-acting β1-blockers, as aimed, in all included studies. In addition to these hemodynamic parameters, Kakihana et al. reported that mean left ventricular ejection fraction (LVEF) was similar between landiolol and control groups [10]. Further, Morelli et al. described that esmolol increased left ventricular stroke work index while it did not affect the right ventricular stroke work index, as well as right atrial pressure and mean pulmonary arterial pressure [6]. Therefore, ultrashort-acting β1-blockers deem to mainly improve the left ventricular efficiency by reducing HR.

**Fig. 1:**
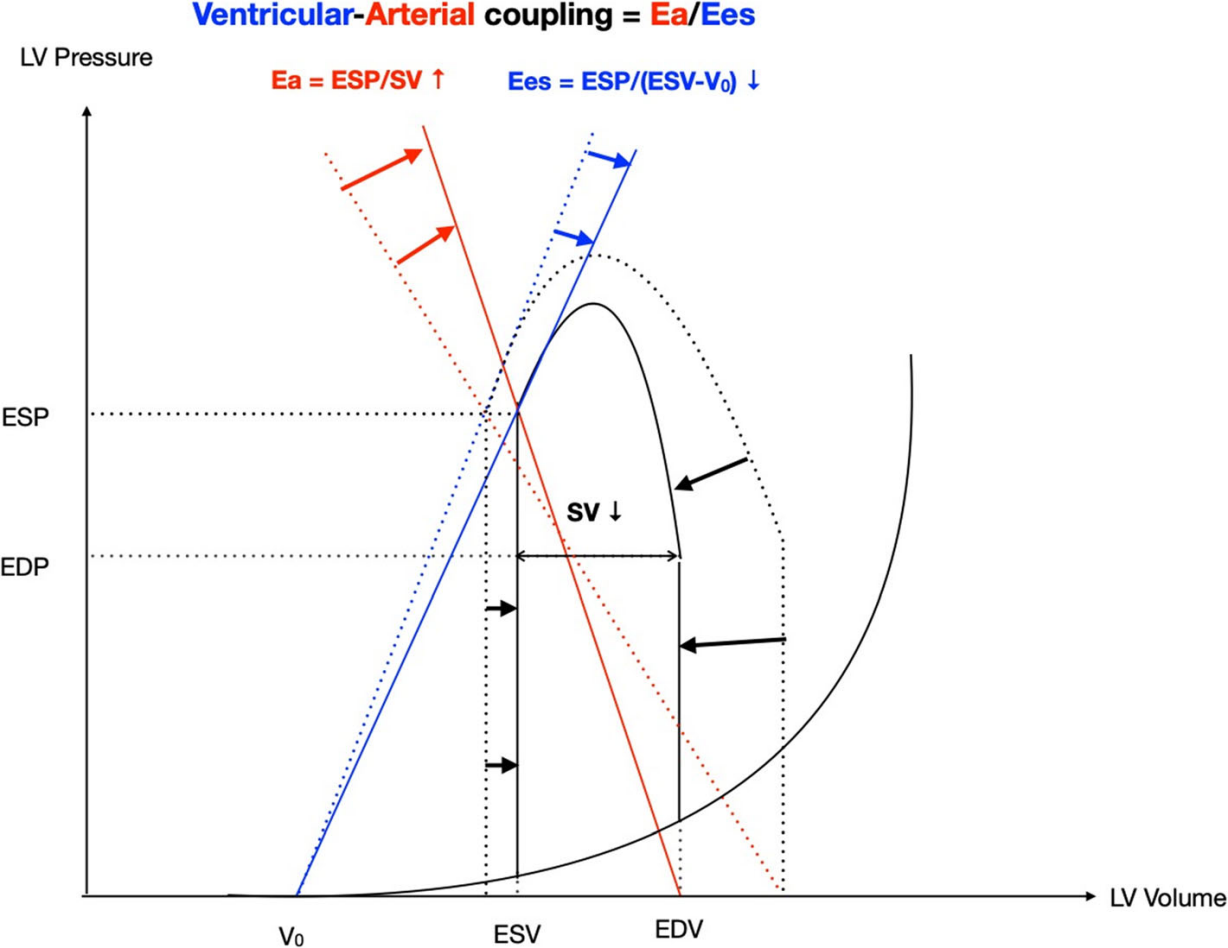
Schematic presentation of ventricular-arterial coupling on the pressure-volume plane. The solid line represents the changes of the pressure-volume relationship, arterial elastance (Ea), and left ventricular end-systolic elastance (Ees) in sepsis, while the dashed lines show the baseline of them. Ea, arterial elastance; Ees, left ventricular end-systolic elastance; ESP, end-systolic pressure; SV, stroke volume; ESV, end-systolic volume; V0, volume intercept of ventricular end-systolic pressure-volume relationship (hypothetical unstressed volume of left ventricle); EDP, end-diastolic pressure; EDV, end-diastolic volume

On the other hand, volume status and vascular resistance can affect cardiac systolic parameters, such as LVEF or SVI. Because β1-blocker affects not only cardiac systolic function but also the afterload, it is important to know how β1-blocker affects an afterload-independent cardiac systolic function. Ventricular-arterial (V-A) coupling defined as the ratio between arterial elastance (Ea) and left ventricular end-systolic elastance (Ees), has recently attracted attention as such an afterload-independent cardiac function [11] (Fig. 1). When Ea/Ees is near unity, cardiovascular efficiency is considered to be optimal. Guarracino et al. reported that septic patients often showed elevated V-A coupling (>1.36), which is called V-A decoupling [12]. Tachycardia in sepsis can decrease stroke volume, resulting in an increased Ea and V-A decoupling. HR reduction with ultrashort-acting β1-blockers reduces Ea, leading to an improvement of cardiovascular efficiency [13]. In patients with sepsis, hyperkinetic status is known to be associated with higher mortality [14]. Hence, the mortality benefit of ultrashort-acting β1-blocker might be explained by suppression of hyperkinetic status by improving cardiovascular efficiency.

On the other hand, many clinicians perhaps concern about medication-induced hemodynamic instability despite evidence of unchanged CI, MAP, and vasopressor requirements. A recent study reported that HR reduction using fast esmolol titration in the very early phase of septic shock was associated with an increased risk of hypotension and decreased CI [15]. This study suggests that ultrashort-acting β1-blockers should be initiated only after completing initial resuscitation. Also, in most studies included in our meta-analysis, patients with severe cardiac dysfunction were excluded. Thus, a cardiac function needs to be evaluated prior to the initiation of β1-blockers.

## Clinical application of β1-blocker in sepsis

Our recent meta-analysis only included septic patients with persistent tachycardia after initial resuscitation [3]. Persistent tachycardia 24 h after adequate fluid resuscitation is an independent risk factor for mortality in patients with sepsis [1], suggesting that the presence of tachycardia itself could impact the prognosis. In addition, medications that can cause tachycardia such as epinephrine or dobutamine require extra caution since they are reported to worsen outcomes [16]. Among studies included in our recent meta-analysis, only one RCT reported types of tachycardia. In this study, the majority (80%) had sinus tachycardia, while the rest was either atrial fibrillation or atrial flutter [10]. On the other hand, many clinicians still do not feel comfortable controlling sinus tachycardia since they believe that the treatment of underlying aetiologies should be prioritized. In the real-world setting, however, it could be difficult to control persistent sinus tachycardia only with treatments for the underlying condition since it takes time for antibiotics and source control for infection to work enough to stabilize HR. Therefore, it still deems to be reasonable to control sinus tachycardia with ultrashort-acting β1-blockers. Given that most of the included studies in our meta-analysis defined persistent tachycardia as HR ≥ 95 bpm despite initial resuscitation after 24 h, ultrashort-acting β1-blockers should be administered to keep HR < 95 bpm only after initial resuscitation for 24 h. However, the duration of ultrashort-acting β1-blockers differs among included studies. Therefore, studies investigating the optimal duration of β1-blocker are still warranted.

## Conclusion

We described the metabolic and hemodynamic effects and clinical evidence of β1-blockers in patients with sepsis. Accumulating evidence is supporting the use of β1-blockers while larger RCTs to clarify the effect of β1-blockers are still warranted.

## Data Availability

Not applicable.

## Abbreviations

HR: Heart rate
RCT: Randomized controlled trial
SVI: Stroke volume index
CI: Cardiac index
MAP: Mean arterial pressure
V-A coupling: Ventricular-arterial coupling
Ea: Arterial elastance
Ees: Left ventricular end-systolic elastance
